# Dosimetric advantage of using 6 MV over 15 MV photons in conformal therapy of lung cancer: Monte Carlo studies in patient geometries

**DOI:** 10.1120/jacmp.v3i1.2592

**Published:** 2002-01-01

**Authors:** Lu Wang, Ellen Yorke, Gregory Desobry, Chen‐Shou Chui

**Affiliations:** ^1^ Department of Radiation Oncology University of Pennsylvania School of Medicine Philadelphia Pennsylvania 19104; ^2^ Department of Medical Physics Memorial Sloan‐Kettering Cancer Center 1275 York Avenue, New York New York 10021

**Keywords:** Monte Carlo, inhomogeneity, lung density, dose uniformity

## Abstract

Many lung cancer patients who undergo radiation therapy are treated with higher energy photons (15‐18 MV) to obtain deeper penetration and better dose uniformity. However, the longer range of the higher energy recoil electrons in the low‐density medium may cause lateral electronic disequilibrium and degrade the target coverage. To compare the dose homogeneity achieved with lower versus higher energy photon beams, we performed a dosimetric study of 6 and 15 MV three‐dimensional (3D) conformal treatment plans for lung cancer using an accurate, patient‐specific dose‐calculation method based on a Monte Carlo technique. A 6 and 15 MV 3D conformal treatment plan was generated for each of two patients with target volumes exceeding 200 cm^3^ on an in‐house treatment planning system in routine clinical use. Each plan employed four conformally shaped photon beams. Each dose distribution was recalculated with the Monte Carlo method, utilizing the same beam geometry and patient‐specific computed tomography (CT) images. Treatment plans using the two energies were compared in terms of their isodose distributions and dose‐volume histograms (DVHs). The 15 MV dose distributions and DVHs generated by the clinical treatment planning calculations were as good as, or slightly better than, those generated for 6 MV beams. However, the Monte Carlo dose calculation predicted increased penumbra width with increased photon energy resulting in decreased lateral dose homogeneity for the 15 MV plans. Monte Carlo calculations showed that all target coverage indicators were significantly worse for 15 MV than for 6 MV; particularly the portion of the planning target volume (PTV) receiving at least 95% of the prescription dose $\left(V_{95}\right)$ dropped dramatically for the 15 MV plan in comparison to the 6 MV Spinal cord and lung doses were clinically equivalent for the two energies. In treatment planning of tumors that abut lung tissue, lower energy (6 MV) photon beams should be preferred over higher energies (15–18 MV) because of the significant loss of lateral dose equilibrium for high‐energy beams in the low‐density medium. Any gains in radial dose uniformity across steep density gradients for higher energy beams must be weighed carefully against the lateral beam degradation due to penumbra widening.

PACS number(s): 87.90.+y, 87.52.–g

## I. INTRODUCTION

Many lung cancer patients who undergo radiation therapy are treated with photons of higher energy (15–18 MV). This is chiefly motivated by the deeper penetration of higher energy photons which, it is argued, provides better dose uniformity in the target. However, this statement does not consider the effects of increased lateral scatter in the low‐density lung. A secondary advantage of using higher energy photons is skin sparing. But with the increasing use of 3‐dimensional conformal radiation therapy (3D‐CRT), including intensity‐modulated techniques (IMRT) with beams incident from different directions over limited surface areas, the need for skin sparing associated with higher photon energy is greatly reduced. Therefore, concerns about the effects of the larger lateral scatter for electrons recoiling from high‐energy photons in lung become prominent. Because these electrons travel farther in lung tissue than in water‐density tissue, their extended lateral spread increases the width of the penumbra and leads to a loss of dose within a beam near its edge. The effects of this loss of lateral electron equilibrium have been reported in the literature^1^ ^–^ ^5^ for both single‐ and parallel‐opposed fields, for low (6 MV) and high (>10MV) beam energies in phantoms. The effect is most pronounced for lower lung densities $\left(0.1‐0.2{g/\text{cm}}^{3}\right)$, higher photon energies and small field sizes.

Lower densities $\left(0.1–0.2{g/\text{cm}}^{3}\right)$ in the normal lung are quite common, especially for patients with concurrent emphysema. Moreover, some studies^6^ ^,^ ^7^ have recommended a deep inspiration breath‐hold (DIBH) technique for lung patients to intentionally reduce lung density during radiation treatment and exclude more normal lung tissue from the treatment beam. Mean density changes achieved by this technique were from 0.26 g/cm^3^ for normal breathing to 0.19 g/cm^3^ for DIBH, for an average decrease of 26%.^7^ Others have found^8^ that lung cancer patients with lower lung densities tend to also have larger lungs, which increases the path length of treatment beams within the lung, making accurate density correction more important.

Most dose calculation algorithms used for treatment planning fail to account for all the lateral scatter effects in lung tissues, even though they make longitudinal corrections such as equivalent pathlength or Batho correction.^9^ They may provide an overoptimistic estimate of dose coverage of a tumor surrounded by lung tissue. This can result in an underdose of the tumor target. There is clinical evidence that small decreases in dose (7–15%) can significantly reduce local tumor control.^10^ ^–^ ^12^ These factors indicate the importance of reexamining the effect of tissue inhomogeneity on dose uniformity using a more accurate dose calculation method, and reevaluating the advantage or disadvantage of using higher versus lower megavoltage energy photons in the conformal radiation therapy of lung cancer. For this purpose, we have performed a dosimetrical comparison between 6 and 15 MV photons in lung cancer conformal treatment planning using an accurate inhomogeneity correction method based on the Monte Carlo (MC) technique. We used the MC dose calculation method^13^ in a “real‐patient” environment to assess the relative success of low energy (6 MV) versus high energy (15 MV) in providing homogeneous dose to tumors in the lung and mediastinum.

## II. METHODS AND MATERIALS

### A. Conventional clinical planning process

We used the planning CT images of two lung cancer patients, each of whom had been scanned in the supine position with his/her arms raised within individually fabricated immobilization casts (Alpha Cardle Molds, Akron, OH). The gross tumor volume (GTV) was outlined by a physician on each of the pertinent images. Margins of 1–1.5 cm were added to the GTV to generate the planning target volume (PTV). The cord and normal lung were also outlined on the images. Both patients had rather large tumors; both GTVs were approximately 98 cm^3^ and the PTVs were 260 cm^3^ and 280 cm^3^. The average field size is $13\times 13{\text{cm}}^{2}$. Experienced planners selected the beam directions and designed the aperture shape to conform to the PTV in the beam's eye view with a 6‐mm margin around the PTV. Each patient's plans utilized four wedged fields. The beam wedges and weights were selected to satisfy the physician's objectives relative to the uniformity of target coverage and normal tissue sparing. The 3D‐CRT plans were initially designed for 6 MV beams on the Memorial Sloan Kettering Cancer Center (MSKCC) treatment planning^14^ system. This system employs a measurement‐based dose‐calculation algorithm that corrects for irregular fields with a pencil‐beam convolution method and corrects for tissue inhomogeneities by computing the equivalent pathlength along the direction of each ray. However, the changes of lateral electron scattering in media differing from water are ignored. For each patient's 6 MV relative isodose plan, the beam weights were adjusted so that the dose to 95% of the PTV $\left(D_{95}\right)$ was 100%. The same beam directions, apertures, and wedges were then used in the 15 MV plans that were also calculated with the MSKCC treatment planning system. The beam weights for 15 MV were set so that the isocenter received the same dose from each beam as in the 6 MV plan. As judged by the isodose distributions and dose‐volume histograms (DVHs) calculated by the treatment planning system, the 6 and 15 MV plans appears suitable for treatment delivery and were quite similar. Indeed, the 15 MV distributions appeared to be slightly superior on the basis of such plan evaluation indices as target dose uniformity and reduction of dose to soft tissues outside of the target.

### B. Monte Carlo calculations

The MC treatment plans were derived from the MC dose calculations^13^ that had been previously benchmarked by experiments in inhomogeneous phantoms.^15^ The method employs the EGS4 system^16^ for the simulation of radiation interactions. Particle transport and energy scoring were handled by the user code MCPAT^13^ The beam information from the 3D‐CRT treatment plans described above (viz. energy, orientation, aperture, and wedge angle) were converted to the format accessible to the MC calculation. An energy spectrum that was derived from phase‐space data and benchmarked by measurement^17^ was used instead of the nominal energy for the specific treatment machine. The incident fluence distribution was obtained by convolution of each beam aperture with the source distribution^13^ The MC algorithm calculated the dose by simulating all of the radiation interactions based on a probability distribution derived from first principles. Charged particles and scattered radiation were traced through the patient geometry as defined by the patient‐specific CT images. These CT images also provided the electron density array by which the particles’ pathlength could be evaluated and therefore dose scoring could be performed. Since the MC method accounted for the variation of local electron density throughout the entire treatment region, the perturbation effects of local tissue heterogeneities were taken into account naturally. Further details on the method are provided in Ref. 13. More than 2000 first interactions were sampled per voxel (cubes of 3‐mm sides) and a statistical uncertainty of 1.0% in the target volume was achieved.

### C. Monte Carlo treatment plans

To generate the MC calculated dose distribution that results from the beams delivered based on conventional calculations, it is necessary to assure that the same monitor units (MU) are applied to both the conventional and the MC plans. Calibration factors relating the MC output of dose per fluence to the dose per MU were established by performing MC calculations for 6 and 15 MV under the calibration conditions. The inverse of the central axis dose per unit fluence at $d_{\max}$ gives the fluence to produce 1 cGy at $d_{\max}$ under the calibration condition ($10\times 10{\;\text{cm}}^{2}$ and source‐to‐axis distance = 100 cm). This is the same as the fluence per MU, given the fact that 1 MU delivers 1 cGy at $d_{\max}$ under the calibration conditions. The MC calculated doses in the unit of dose per fluence can be converted to the dose per MU by multiplying this factor–our calibration factor. Each conventional percent isodose plan was converted to an absolute dose plan with 100 cGy delivered by the 100% isodose line. For each beam in the corresponding MC plan, the MC‐calculated dose array was then multiplied by the product of the conventional plan beam weight and the energy‐dependent calibration factor.

The following aspects of the 6 and 15 MV plans for each patient were compared: (i) isodose distributions; (ii) for the PTV and GTV dose‐volume histograms and dose parameters of mean dose, $D_{95}$ (lowest dose encompassing 95% of the target) and $V_{95}$ (volume receiving 95% of the dose or more); and (iii) for cord and normal lung: DVH and dose parameters $D_{05}$ (lowest dose received by the hottest 5% of the volume) and $D_{\max}$ (maximum dose).

## III. RESULTS

### A. Dose distributions for single field

**Figure 1:**
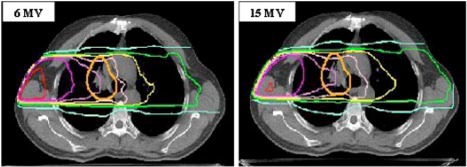
(Color) Isodose distributions from a single right‐lateral (wedged) field only. The thicker line represents the PTV The MUs for each beam were set to deliver 60 cGy to isocenter. The isodose levels displayed are 90, 70, 60, 50, 40, 30, and 10 cGy The graph on the left represents the isodose distribution for 6 MV, and the right one for 15 MV photons.

To better understand the difference between the MC results for the 6 and 15 MV treatment plans we first compared the MC calculated dose distributions of a single treatment field. Figure 1 shows the dose distributions in the transverse plane through the isocenter of the right lateral field (with a 30° wedge) for Patient No. 1. Since the plans of both patients showed similar dependence on beam energy, the isodose distributions of Patient No. 1 will be presented here as typical. The 6 MV distribution is at the left and 15 MV distribution is at the right. In order to demonstrate the differences in the absolute dose distribution, the number of monitor units for each energy was chosen to deliver 60 cGy to the isocenter. The PTV is shown as a thicker orange line. The isodose lines represent the absolute doses in centigrey. The expected features predicted by standard calculation algorithms are clearly seen; there is increased penetration of the 15 MV beam into the contralateral lung (e.g., 40 and 30 cGy lines) and reduced dose to soft tissue at the entrance side (e.g., the 6 MVbeam has a noTable 90 cGy “hot spot” and the 15 MV has a small one). However, features related to lateral disequilibrium are also seen. The penumbra of the 15 MV beam in the low‐density lung undergoes more pronounced spreading than the 6 MV beam. Furthermore, for the 15 MVbeam, the 60 cGy, and 50 cGy isodose lines “collapse” toward the beam center, providing inferior coverage of the PTV, while the low (e.g., 10 cGy) lines expand outward in the low‐density lung tissue. These effects, while present at 6 MV, are less pronounced.

### B. Plan isodose distribution

**Figure 2:**
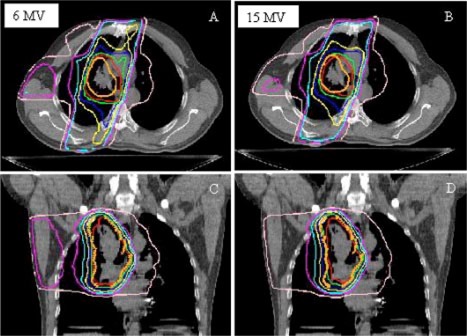
(Color) Composite isodose distributions of four‐field treatment plans for Patient No. 1 from the transverse (A,B) and coronal (C,D) views, for 6 and 15 MV. The thicker line and dots represent the PTV The isodose levels of 95%, 90%, 80%, 70%, 50%, 40%, and 20% are shown.

Figure 2 compares the percent isodose distributions (generated by setting 100%=100 cGy) for the MC‐calculated 6 MV plan and 15 MV plan for Patient No. 1. Comparing the PTV coverage of the plans in the transverse cuts through isocenter [(Figs. 2A) and (2B)], the 95% isodose line (in red) for 15 MV beam [(Fig. 2B)] shrinks into the PTV, especially on the right side where the PTV is within lung. The MC‐calculated 6 MV plan [(Fig. 2A)] with the same apertures keep the 95% line outside the PTV. On the coronal slice through isocenter [(Figs. 2C) and (2D)], the 95% line in the 15 MV plane [(Fig. 2D)] covers both superior and inferior aspects of the PTV poorly. The same feature is seen on the sagittal view (not shown). The isodose distribution near the cord is about the same for both 6 and 15 MV. This makes sense for this patient, since on the transverse slice, the patient's back looks quite fleshy and the cord is surrounded by water–equivalent tissue and bone. It is the increased penumbra in the low‐density region with higher photon energy that has resulted in the shrinkage of the higher isodose line.

### C. Dose‐volume histograms of PTV

**Figure 3:**
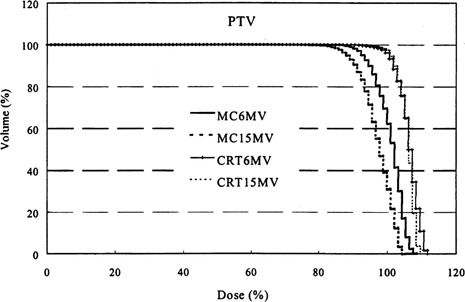
Comparison of the dose‐volume histograms of the PTV for Patient No. 1 using 6 and 15 MV photon beams, using both the treatment planning system (CRT) and the Monte Carlo method (MC).

A comparison of DVHs for the PTV of Patient No. 1 using the MC method is shown in Fig. 3. The mean doses for the two energies do not differ dramatically (100.5% for 6 MV and 96.8% for 15 MV), but the dose coverage indices, $D_{95}$ and $V_{95}$, differ more significantly. $D_{95}$ is 92.3% for 6 MV and 87.8% for 15 MV, and $V_{95}$ is 88.1% for the 6 MV plan and 67.8% for the 15 MV plan. The steep slope of the integral DVH in the high‐dose region makes the volume covered by a particular dose level a sensitive indicator of the difference between the two plans. It is apparent from the $V_{95}$ indicator that the PTV dose uniformity is better with the 6 MV plan than with the 15 MV plan. For a direct visual comparison between the results of MC calculation and clinical expectations based on conventional calculations (the pencil beam algorithm using the equivalent pathlength method), the DVHs for the PTV of Patient No. 1 from the treatment planning system are also presented in Fig. 3. Using conventional calculations, the 6 and 15 MV plans seemed quite similar as judged by the isodose distributions (not shown) and dose‐volume histograms. In fact, the two plans have almost identical DVHs, with $V_{95}$ being 99% and $D_{95}$ being 100% for both energies. Furthermore, the 15 MV distribution appeared to be slightly superior on the basis of such plan evaluation indices as target dose uniformity and reduction of dose to soft tissues outside of the target. However, when tissue inhomogeneity effects are computed more accurately, as with the MC calculation, the 15 MV distributions are no longer superior than those of 6 MV beam. Moreover, both $V_{95}$ and $D_{95}$ are lower than expected. This effect is more pronounced for the higher energy, as seen in Fig. 3. The results for Patient No. 2 were qualitatively similar. For this patient, at 6 MV, the mean dose, $D_{95}$ and $V_{95}$ were 100.8%, 95%, and 94.9%, respectively, while for 15 MV, the corresponding indices were 97.4%, 89.7%, and 78.4%. Such differences could be clinically significant if the decision to use higher energy is based on the dose distribution predicted by a treatment planning system that does not account for lateral scatter effects.

### D. Dose‐volume histograms of the GTV

**Figure 4:**
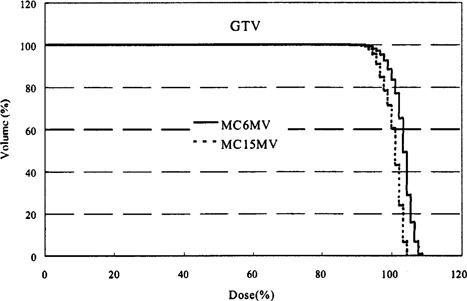
Comparison of the dose‐volume histograms of the GTV for Patient No. 1 using 6 and 15 MV photon beams and the Monte Carlo dose‐calculation method.

For the GTV, the 15 MV coverage indices are not significantly worse than the corresponding 6 MV indices, unlike what is observed for the PTV. This is because of the large distance (1.6 cm) from GTV edge to aperture edge in beam's‐eye view. Figure 4 compares the 6 MV and 15 MV DVHs of the GTV for Patient No. 1. For this patient, $D_{95}$ is 98.6% for 6 MV and 94.6% for 15 MV, and $V_{95}$ is 97.7% for the 6 MV plan and 93.6% for the 15 MV plan. For Patient No. 2, the results are similar, with $D_{95}$ being 98.6% for 6 MV and 96.2% for 15 MV, and $V_{95}$ being 100.0% versus 97.8% for 6 and 15 MV, respectively. Thus, the differences in indices are well within 2–4%. The dose received by the tumor during treatment is likely, however, to be lower than indicated by the GTV dose‐volume statistics as setup errors and microscopic extension of disease causes tumor tissue to occupy the space between GTV and PTV at least part of the time. Although increasing the margin from PTV to aperture edge would give better PTV coverage, it would have the undesirable effect of increasing normal tissue toxicity of lung.

### E. Dose‐volume histograms of critical structures

**Figure 5:**
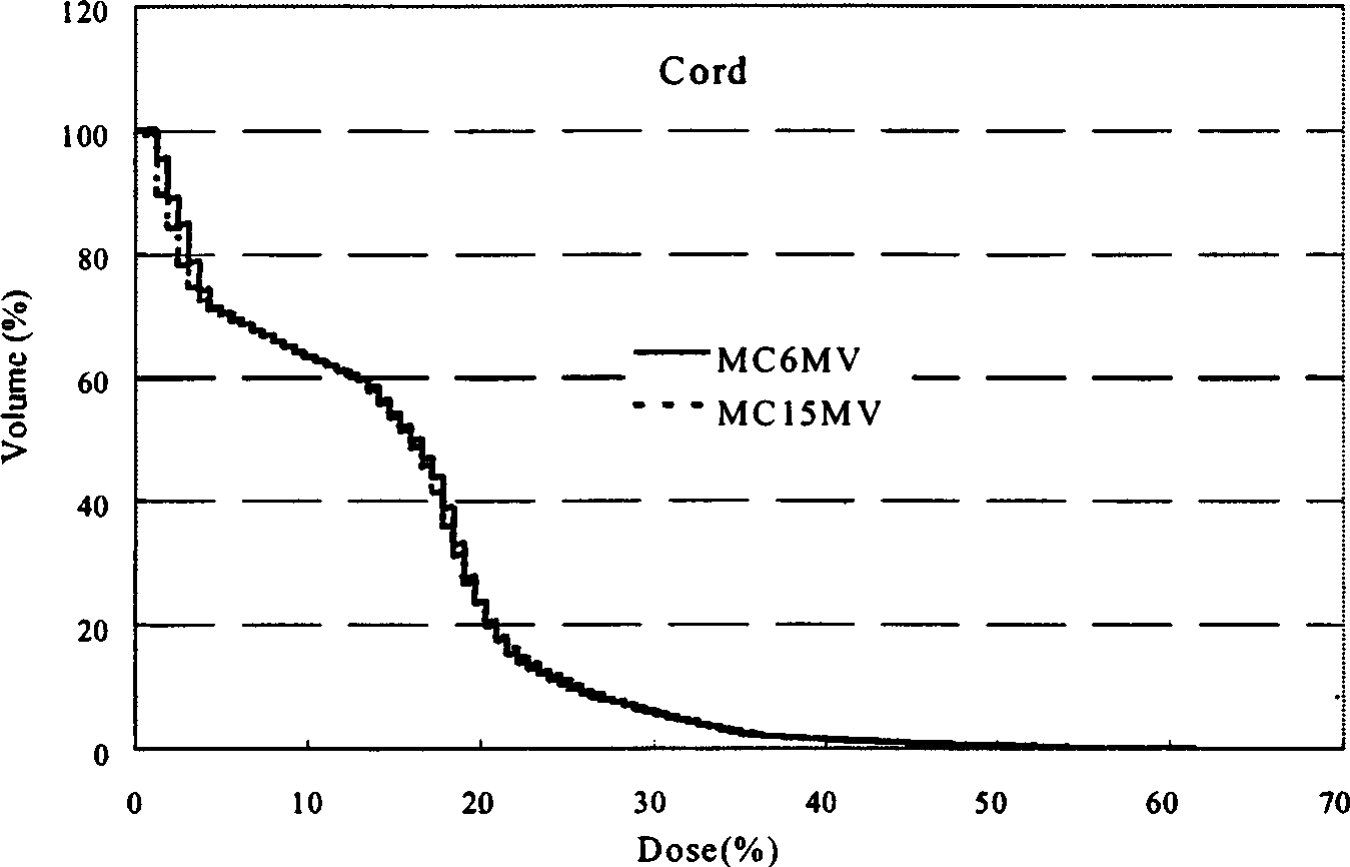
Comparison of the dose‐volume histograms of the cord for Patient No. 1 using 6 and 15 MV photon beams and the Monte Carlo dose‐calculation method.

The dose limiting critical structures in lung cancer treatment are the lung and the spinal cord. Spinal cord complications are sensitive to high‐dose regions, even if they involve a small volume while the severe lung complication of radiation pneumonitis is strongly dependent on the volume of significantly irradiated tissues. Figure 5 compares the DVH of the cord for Patient No. 1 from the two Monte Carlo plans using different photon energies. It is seen that the two plans deliver essentially the same dose distribution to cord with the 15 MV plan having a slightly lower cord maximum–59% for 15 MV versus 61% for 6 MV The $D_{05}$ (the dose to the hottest 5% of the volume) is approximately the same for 6 and 15 MV Similar results were found for Patient No. 2.

**Figure 6:**
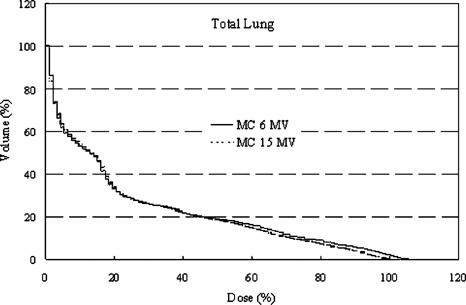
Comparison of the dose‐volume histograms of the total lung for Patient No. 1 using 6 and 15 MV photon beams and the Monte Carlo dose‐calculation method.

For the total lung volume, the DVHs of the two plans are similar, with the 15 MV plan showing a slight improvement over the 6 MV plan, as seen in Fig. 6. The slightly higher maximum dose to lung observed for the 6 MV plan reflects the wider high‐dose region compared to the high‐energy plan. The larger volume in the DVH low‐dose range for the 15 MV plan results from the more penetrating quality of the beam.

## IV. CONCLUSIONS

When passing through a low‐density medium such as lung, the penumbra of a 15 MV photon beam is broadened more than that of a 6 MV beam, although decreased attenuation of the higher energy beam gives a more homogeneous dose distribution in the downstream direction. The lateral spreading of the high‐energy beams in lung is seldom accounted for in treatment planning system dose calculations, although many standard techniques have been developed to accurately handle attenuation in inhomogeneous media. Lateral disequilibrium has the clinically undesirable effect of reducing the quality of PTV coverage by high‐energy beams relative to lower energy beams even if both energies are delivered through the same portals and are weighted to give a similar dose distribution according to conventional calculations. Two cases were considered for this study. In both cases the PTV exceeded 250 cm^3^ in volume and neither patient had exceptionally low‐density lungs. In the 15 MV plans, MC calculation showed that the dose enclosing 95% of the PTV volume $\left(D_{95}\right)$ was approximately 10% lower than expected from the treatment planning system for Patient No. 1 and 13% lower for Patient No. 2. For 6 MV photons, the discrepancy between the expected $D_{95}$ and $D_{95}$ calculated with the more accurate MC method was approximately 5% and 8% for the same two patients. In both cases, the negative effects of lateral disequilibrium in the PTV outweighed the benefits of using higher energy beams to obtain a nominally more homogeneous dose throughout the lung volume.

The extended lateral range of electrons in low‐density media is difficult to compute accurately with a treatment‐planning algorithm that is fast enough to be clinically convenient. The Monte Carlo method used here to revisit conformal multibeam lung treatment plans provides a quantitatively correct estimate of both the lateral and longitudinal effects. The interplay of these two effects is best studied with a Monte Carlo algorithm interfaced to the clinical treatment planning system, but few clinical sites have this capability. Our results support the recommendation of the Radiation Therapy Oncology Group (RTOG) 91‐05 protocol for nonsmall cell lung cancer that radiation therapy treatment should be given with photon energies in the range of 4–12 MV.^18^
